# EEG and ECG based response predictors in depression: Time for personalised medicine or treatment stratification?

**DOI:** 10.1192/j.eurpsy.2021.40

**Published:** 2021-08-13

**Authors:** M. Arns

**Affiliations:** Research Institute Brainclinics, Brainclinics Foundation, Nijmegen, Netherlands

## Abstract

In depression (MDD) treatment there is a clear need for novel treatments, biomarkers and individualized treatment approaches. One of the most promising and most widely investigated biomarkers for antidepressant treatments is the EEG. Most EEG biomarkers however, still lack robustness and reproducibility and suffer significant publication bias as highlighted in a recent meta-analysis (Widge et al., 2018). Therefore, large controlled validation studies are needed with a focus on robustness, replication and clinical relevance. In this presentation results will be presented from the largest EEG Biomarker study to date, the international Study to Predict Optimized Treatment in Depression (iSPOT-D), where 1008 MDD patients were randomized to Escitalopram, Sertraline and Venlafaxine. Drug-class specific (Arns et al., 2016) and drug-specific (Arns, Gordon & Boutros, 2015) biomarkers will be highlighted as well as preliminary data from a prospective feasibility trial. Furthermore, data will be presented on repetitive Transcranial Magnetic Stimulation (rTMS) treatment in MDD on EEG and clinical predictors (Krepel et al., 2018; 2019) and a new method called Neuro-Cardiac-Guided TMS (NCG TMS), that exploits network connectivity in the frontal vagal pathway, as a target engagement approach (Iseger et al., 2019). Finally, clinical implications and implementations will be discussed from a ‘treatment stratification’ perspective, which might be a more realistic goal relative to ‘personalized medicine’ perspective.

**Disclosure:**

MA is unpaid research director of the Brainclinics Foundation, a minority shareholder in neuroCare Group (Munich, Germany), and a co-inventor on 4 patent applications related to EEG, neuromodulation and psychophysiology, but receives no royalties related

